# Omega-3 nutraceuticals, climate change and threats to the environment: The cases of Antarctic krill and *Calanus finmarchicus*

**DOI:** 10.1007/s13280-020-01472-z

**Published:** 2021-01-27

**Authors:** Alfonso Prado-Cabrero, John M. Nolan

**Affiliations:** Nutrition Research Centre Ireland, School of Health Science, Carriganore House, Waterford Institute of Technology, West Campus, Carriganore, Waterford, Ireland

**Keywords:** Antarctic krill, *Calanus finmarchicus*, Climate change, Docosahexaenoic acid, Eicosapentaenoic acid, Krill oil

## Abstract

The nutraceutical market for EPA (eicosapentaenoic acid) and DHA (docosahexaenoic acid) is promoting fishing for *Euphasia superba* (Antarctic krill) in the Southern Ocean and *Calanus finmarchicus* in Norwegian waters. This industry argues that these species are underexploited, but they are essential in their ecosystems, and climate change is altering their geographical distribution. In this perspective, we advocate the cessation of fishing for these species to produce nutraceuticals with EPA and DHA. We argue that this is possible because, contrary to what this industry promotes, the benefits of these fatty acids only seem significant to specific population groups, and not for the general population. Next, we explain that this is desirable because there is evidence that these fisheries may interact with the impact of climate change. Greener sources of EPA and DHA are already available on the market, and their reasonable use would ease pressure on the Arctic and Antarctic ecosystems.

## Introduction

The last years have seen an increase in the popularity of fish oil nutraceuticals (Kantor et al. 2016). These oils, rich in the omega-3 fatty acids EPA (eicosapentaenoic acid) and DHA (docosahexaenoic acid), promise to improve cardiovascular health and cognition, as well as protect against diabetes or even cancer (Shahidi and Ambigaipalan 2018). However, voices have been raised denouncing that an increase in the catches of forage fish to supply this growing industry is putting pressure on the already decimated fishing grounds of the planet. Jenkins and colleagues described this threat and put it into the perspective of the studies supporting the health benefits of these oils, and concluded that these fisheries might not be justified (Jenkins et al. 2009).

Various companies, in search of alternative sources of EPA and DHA, are now exploiting zooplankton species. They call this practice ‘fishing down the food web’, and justify their activity in the high biomasses of these species in the oceans. Thus, catches of *Euphasia superba* (known as Antarctic krill), once used as food for domestic animals or even as fertiliser (Nicol 2018), are now increasing in the Southern Ocean to prepare these capsules. In addition, a new commercial fishery has recently started in Norwegian waters to capture *Calanus finmarchicus*, a 2–4 mm long calanoid copepod with long antennae and a reddish torpedo-shaped body (Norwegian Directorate of Fisheries 2019).

The companies exploiting Antarctic krill and *C. finmarchicus* claim that these species are underexploited. However, both zooplankton species are essential in their ecosystems, as they occupy the intermediate level in their respective trophic webs, where they link primary producers with predators (Fauchald et al. 2011; Atkinson et al. 2014). Climate change is affecting the stocks and geographic distribution of these zooplankton species (Beaugrand et al. 2003; Flores et al. 2012b; Atkinson et al. 2019), and in the case of Antarctic krill, there is a debate about detrimental effects of the fishery (Krüger et al. 2020; Watters et al. 2020).

In this perspective, we defend that exploiting Antarctic Krill and *C. finmarchicus* to prepare EPA and DHA nutraceuticals is not rational. To do this, we review the current science available on the cardiovascular and cognitive benefits of these fatty acids, and we conclude that the general population does not benefit from consuming these oils. This, together with the threats posed by these fisheries along with climate change to exploited ecosystems, make us propose the cessation of fishing for these zooplankton species.

## Health benefits of EPA and DHA

### Based on the best available evidence, there are not significant cardiovascular benefits of EPA and DHA in the broad population

**Fig. 1:**
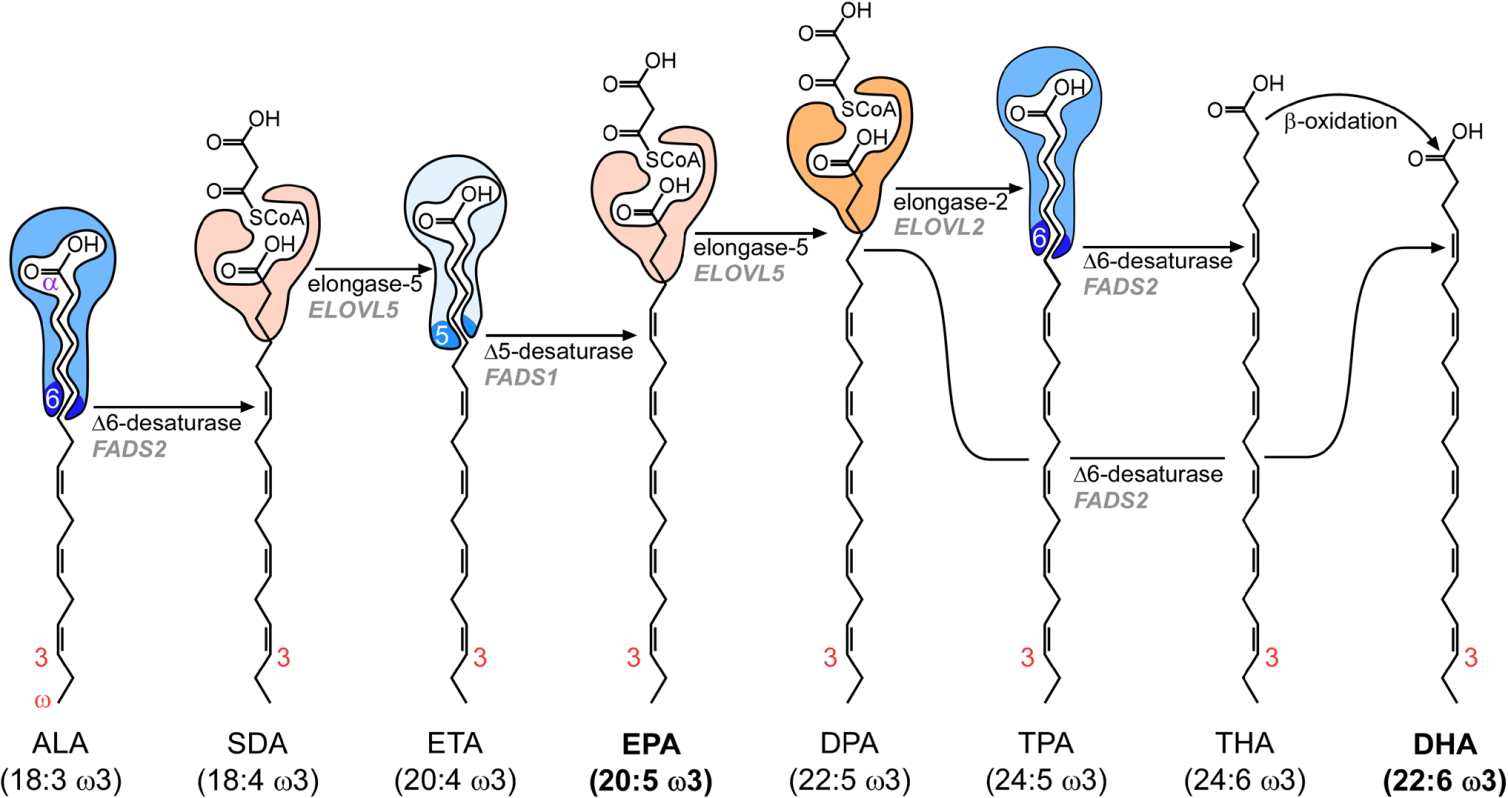
Biosynthesis pathway of EPA (eicosapentaenoic acid) and DHA (docosahexaenoic acid) in the liver. The pathway starts with the essential fatty acid ALA (α-linolenic acid), which must be obtained from the diet. This fatty acid undergoes a series of desaturations performed by Δ5-desaturase and Δ6-desaturase (*FADS2* and *FADS1* genes, respectively), and also elongations, performed by elongase-5 (*ELOVL5* gene) to yield EPA. Then, EPA is elongated by elongase-5 to yield DPA. From DPA, DHA can be produced either by elongation (elongase-2, *ELOVL2* gene), desaturation (Δ6-desaturase) and β-oxidation in the peroxisome, or by direct desaturation by Δ6-desaturase (Park et al. 2015). Apart from DHA, EPA and DPA (docosapentaenoic acid) are found in appreciable amounts in human plasma. The alpha (α) end of the fatty acids denotes the carbon counting start from the carboxy end and coincides with the recognition site of the desaturase enzymes, whose number specifies the carbon where the desaturation is performed. The omega end denotes the end of the molecule opposite to the carboxy group and is used to denote the position of the desaturations in the molecule. *SDA* stearidonic acid, *ETA* eicosatetraenoic acid, *TPA* tetracosapentaenoic acid; THA, tetracosahexaenoic acid

EPA and DHA are long-chain (≥ C_20_) polyunsaturated fatty acids (LC-PUFA) of the omega-3 family. Humans obtain these fatty acids ingesting fish and seafood, but we are also genetically equipped to produce these fatty acids from dietary α-linolenic acid (ALA, Fig. 1) (Bradbury 2011). Interest on the benefits of EPA and DHA on human health began when Danish researchers Bang and Dyerberg associated the low incidence of ischemic heart disease in the Inuit people of Greenland to their diet of fish, whales and seals, rich in these fatty acids (Dyerberg et al. 1975; Bang et al. 1976).

This work and the epidemiological studies that followed suggested that EPA and DHA prevent cardiovascular risk (Simopoulos 2002). Despite the importance of these works, we must emphasize that by their nature, these studies are only designed to generate work hypotheses, and not to confirm them (Greenberg 2018). Subsequent randomised placebo-controlled trials (RPCTs), whose design allows questioning the working hypothesis, are yielding, nevertheless, contradictory results. On the one hand, supplementation with EPA and DHA reduced the risk of sudden cardiac death (GISSI-Prevenzione Investigators 1999), slightly reduced death rate and admission to hospital for cardiovascular reasons (Tavazzi et al. 2008), prevented non-fatal coronary events in hypercholesterolaemic subjects (Yokoyama et al. 2007), and lowered the risk of cardiovascular death in subjects with elevated triglyceride levels, but in this case, using only high doses of EPA-ethyl ester (Bhatt et al. 2018). On the other hand, other RPCTs reported that supplementation did not reduce the rate of cardiovascular events in subjects at high risk of cardiovascular events (Bosch et al. 2012), did not lower cardiovascular mortality and morbidity on subjects with multiple cardiovascular risk factors (Roncaglioni et al. 2013), did not decrease the risk of serious cardiovascular events in patients with diabetes and without cardiovascular disease (Bowman et al. 2018) and did not reduce the incidence of major cardiovascular events in patients free of cardiovascular disease (Manson et al. 2019). EPA and DHA industry representatives are concerned about these inconsistencies. Among the possible causes, they point to the use of inadequate statistics, or the general lack of measurement of the levels of EPA and DHA in the participants of the trials, as the researchers do not know the baseline status for these fatty acids or the effectiveness of the formulations in the participants (Rice et al. 2016; Serini and Calviello 2020).

Despite this mix of positive and non-significant results, EFSA recommends the consumption of between 100 and 450 mg of EPA and DHA daily for children, adults and pregnant women (EFSA Panel on Dietetic Products 2010) and allows companies to claim in their products that EPA and DHA contribute to the normal function of the heart (EFSA Panel on Dietetic Products and Allergies 2010b,  2011). FDA, on the other hand, while also allows for health claims, enforces discretion requesting the addition of the statement: “However, FDA has concluded that the evidence is inconsistent and inconclusive” (Center for Food Safety and Applied Nutrition (CFSAN) 2014).

To date, meta-analyses on available RPCTs with EPA- and DHA-rich oils or foods, conducted by independent groups, in different years and with different methodologies, coincide in that EPA and DHA supplementation does not prevent cardiovascular disease (Rizos et al. 2012; Aung et al. 2018; Abdelhamid et al. 2020) (Table 1). The most recent of these meta-analyses included 86 RPCTs with adults at different cardiovascular risks, and concluded that EPA and DHA supplementation did not significantly lower all-cause mortality, cardiovascular mortality, cardiovascular events, stroke or arrhythmia in adults at cardiovascular risk (Abdelhamid et al. 2020). Nevertheless, these authors found low-certainty evidence that supplementation slightly reduced events and mortality from coronary heart disease. These reductions, measured as number needed to treat for an additional beneficial outcome (NNTB) were, however, very small. As an example, these authors estimated that 334 people would need to take EPA and DHA supplements for four years for one person to avoid death from coronary heart disease; the other 333 people would not get any benefit. Given these results, Abdelhamid and colleagues suggest that EPA and DHA supplementation “is probably not useful for preventing or treating cardiovascular disease”. For krill oil, the only meta-analysis of RPCTs performed to date showed similar results to fish oil (Ursoniu et al. 2017). Regarding Calanus oil, the antioxidant, anticholesterolemic and anti-inflammatory roles reported for this oil at the preclinical level (Gasmi et al. 2020) have only been recently tested in humans (Wasserfurth et al. 2020). In this study, the consumption of Calanus oil in people who were exercising moderately contributed to losing weight in a similar way as eating a healthy diet.

**Table 1:** Meta-analyses of the effects of randomised placebo-controlled trials (RPCTs) with EPA and DHA supplementation on cardiovascular and cognitive health

Meta-analysis	Health field	RPCTs analysed	Author’s conclusions
Rizos et al. (2012**)**	Cardiovascular Health	20	Omega-3 PUFA supplementation was not associated with a lower risk of all-cause mortality, cardiac death, sudden death, myocardial infarction, or stroke based on relative and absolute measures of association
Aung et al. (2018**)**	Cardiovascular Health	10	Omega-3 fatty acids had no significant association with fatal or nonfatal coronary heart disease or any major vascular events. It provides no support for current recommendations for the use of such supplements in people with a history of coronary heart disease
Abdelhamid et al. (2020**)**	Cardiovascular Health	86	Moderate- and low-certainty evidence suggests that increasing LCn3 slightly reduces risk of coronary heart disease mortality and events, and reduces serum triglycerides (evidence mainly from supplement trials)
Brainard et al. (2020**)**	Cognition in healthy adults	38	Long-chain omega-3 probably has little or no effect on new neurocognitive outcomes or cognitive impairment
Shulkin et al. (2018**)**	Childhood psychomotor and visual development	38	n–3 PUFA supplementation improves childhood psychomotor and visual development
Chang et al. (2018**)**	ADHD	7	We provide strong evidence supporting a role for n3-PUFAs deficiency in ADHD, and for advocating n-3 PUFAs supplementation as a clinically relevant intervention in this group, especially if guided by a biomarker-based personalisation approach
Liao et al. (2019**)**	Depression	26	Current evidence supports the finding that omega-3 PUFAs with EPA ≥ 60% at a dosage of ≤ 1 g/d would have beneficial effects on depression. We note that the long-term efficacy and health effects of omega-3 PUFA supplementation in depression have yet to be elucidated
Deane et al. (2019**)**	Depression and anxiety	31	Long-chain omega-3 supplementation probably has little or no effect in preventing depression or anxiety symptoms
Canhada et al. (2018**)**	Alzheimer’s disease	7	The effects of omega-3 fatty acids supplementation in mild AD corroborate epidemiologicalobservational studies showing that omega-3 fatty acids may be beneficial in disease onset, when there is slight impairment of brain function
Burckhardt et al. (2016**)**	Dementia	3	We found no convincing evidence for the efficacy of omega-3 PUFA supplements in the treatment of mild to moderate AD

**Fig. 2:**
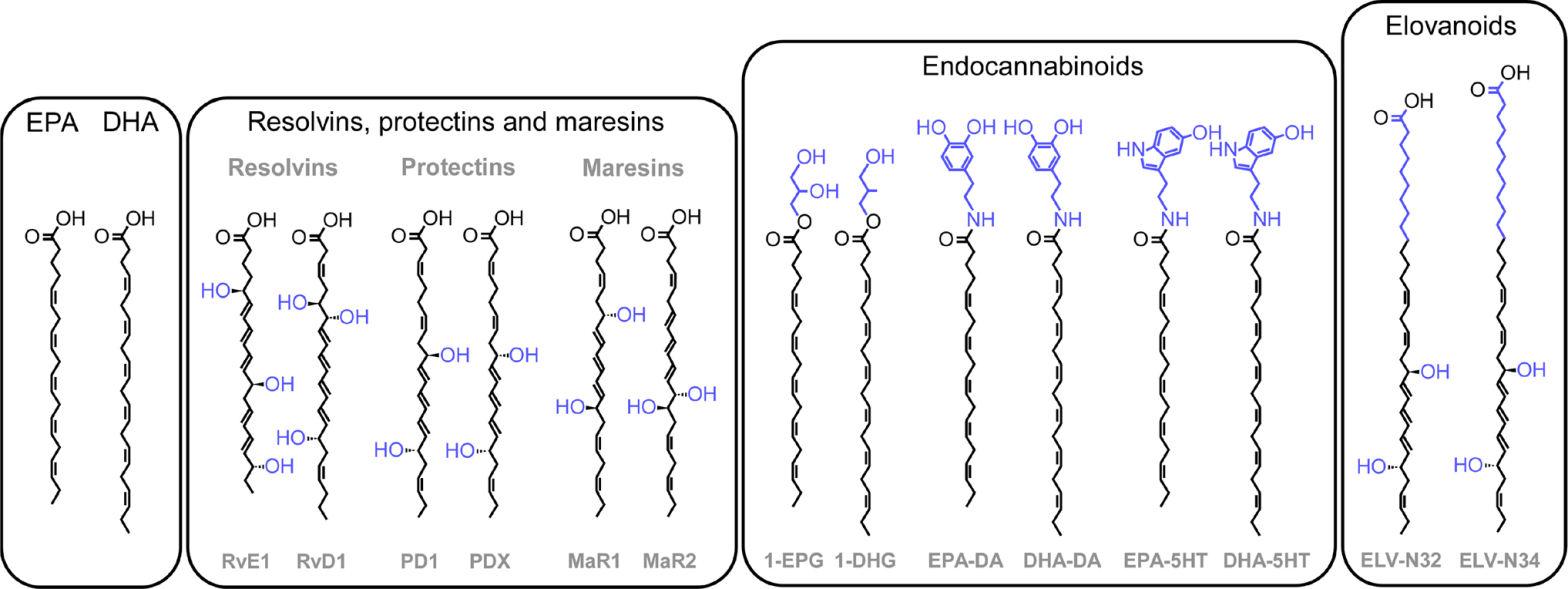
Signalling molecules derived from EPA and DHA. In black is depicted the structure of EPA and DHA. In blue are highlighted the enzymatically made additions yielding resolvins, protectins, maresins, endocannabinoids and elovanoids. *EPA* eicosapentaenoic acid, *DHA* docosahexaenoic acid, *RvE1* Resolvin-EPA 1, *RvD1* Resolvin-DHA-1, *PD1* Protectin-DHA 1, *PDX* Protectin-DHA X, *MaR1* Maresin 1, *Mar2* Maresin 2, *1-EPG* 1-eicosapentaenoyl-glycerol, *1-DHG* 1-docosahexaenoyl-glycerol, *EPA-DA* EPA-Dopamine, *DHA-DA* DHA-Dopamine, *EPA-5HT* EPA-Serotonin, *DHA-5HT* DHA-Serotonin, *ELV-N32* Elovanoid N32, *ELV-34* Elovanoid N34

Importantly, to give full meaning and credibility to the health benefits of a nutrient, it is necessary to describe the molecular mechanisms that underlie the effects found in the clinic. To date, a large number of molecular mechanisms by which EPA and DHA are involved in human physiology have been described. Both fatty acids bind to the GPR120 receptor to promote healthy adipogenesis, maintain insulin sensitisation and control inflammation (Oh et al. 2010; Hilgendorf et al. 2019). EPA and DHA also undergo enzymatic modifications to yield signals such as protectins, resolvins and maresins (Fig. 2) (Watson et al. 2019), which promote resolution of inflammation and modulate the immune response (Spite et al. 2014; Serhan and Levy 2018). EPA and DHA are also precursors of omega-3 endocannabinoids (Fig. 2), which again regulate inflammation (McDougle et al. 2017) but are also involved in cognition, pain and cancer (Watson et al. 2019). Many of these functions of EPA- and DHA-derived molecules have been proposed to mediate the claimed benefits of supplementation. For example, EPA and DHA have been proposed to regulate atherosclerosis (Zehr and Walker 2018), hypertriglyceridemia (Arca et al. 2018), platelet function (Lagarde et al. 2018) and blood pressure (Guo et al. 2019). Of note, some of these EPA- and DHA-derived molecules are even used as a model to synthesize analogues with potentially greater beneficial effects (Imig et al. 2010). Nevertheless, only clear and consistently positive results from human trials, comparable to those published with cell cultures and animal models, can support the use of EPA and DHA to improve cardiovascular health (Mason et al. 2020).

### EPA and DHA supplementation seem to benefit vision and cognition, but in specific cases

EPA and DHA are also claimed as nutrients with beneficial effects in vision and cognition, and much attention is currently being paid to the potential role of these molecules in neurodegenerative disorders, both at the preclinical and at the clinical level (Martins et al. 2020; Yde Ohki et al. 2020). DHA is present in approximately 50% of the phospholipids forming the membranes of the rod photoreceptors in the retina (Shindou et al. 2017), and constitutes about 18% of the total fatty acids present in the grey matter of the brain (Skinner et al. 1993). The importance of DHA in vision and cognition is in great part because DHA-phospholipids enhance the flexibility of specialized cell membranes in these organs and facilitate the function of embedded proteins. This is because DHA is almost entirely populated with double bonds (Fig. 1), which give the entire molecule an unusually high degree of torsional rotation (Gawrisch et al. 2003) that translates in great flexibility in all three axes of space (Barelli and Antonny 2016). Thus, in staked discs of rod photoreceptors, which are composed of highly bent membranes (Burgoyne et al. 2015), DHA-phospholipids facilitate continuous and efficient disc formation and recycling (Shindou et al. 2017). At the same time, in these membranes, DHA-phospholipids assist rhodopsin in its transition from the inactive to the active state when it receives a light photon (Salas-Estrada et al. 2018). In the membrane of neuronal axons (Yang et al. 2012), DHA-phospholipids facilitate the formation of vesicles with neurotransmitters, essential for synaptic transduction (Manni et al. 2018). In addition to these structural roles, DHA, together with EPA, is a precursor in brain and retina of yet another family of signalling molecules, the elovanoids (Fig. 2), which are elongated and hydroxylated derivatives of these fatty acids (Shindou et al. 2017). Elovanoids promote cellular pathways to counteract uncompensated stresses in these organs (Shindou et al. 2017; Do et al. 2019).

These molecular roles of EPA and DHA in vision and cognition suggest clinical benefits for supplementation, at least in certain conditions. Thus, in preterm infants, a meta-analysis suggested that supplementation is beneficial in terms of visual acuity and cognitive development (Shulkin et al. 2018), likely because dietary EPA and DHA compensated the lack of placental transfer of these fatty acids due to premature birth (Larqué et al. 2011).

At the perinatal stage, when the formation of neural circuits is intense, EPA and DHA are likely crucial. As an example, optimal levels of EPA and DHA are essential for the appropriate development of the neural networks that support the reward system in rats (Ducrocq et al. 2020). Congruently, people with major depressive disorder, schizophrenia, or bipolar disorder suffer from low motivation and generally have low levels of these fatty acids in blood (Messamore and McNamara 2016). Interestingly, these low levels of EPA and DHA do not appear to be related to diet (Noaghiul and Hibbeln 2003; Peet 2004).

Children and adolescents with attention deficit hyperactivity disorder (ADHD) significantly improved their clinical symptoms and cognitive performance (Chang et al. 2018), especially individuals with low basal levels of these fatty acids (Chang et al. 2019). Beneficial effects in depression is disputed (Deane et al. 2019; Liao et al. 2019). Alzheimer's patients, who usually have low levels of DHA in the brain (de Wilde et al. 2017) due to a decrease of DHA synthesis in the liver (Astarita et al. 2010), an impairment of DHA import to the brain (Ochiai et al. 2019) and poor eating habits (Samadi et al. 2019), could benefit at the early stage of the disease (Canhada et al. 2018). It is therefore tempting to hypothesise that these mental disorders may have a genetic component responsible of a reduced synthesis of EPA and/or DHA, which are essential for the successful development, maintenance and function of neural networks.

Nevertheless, a recent meta-analysis of 38 RPCTs concluded that supplementation does not seem to help healthy adults, as it does not prevent the onset of neurocognitive illness (Brainard et al. 2020). Despite this, EFSA allows the industry to claim that supplementation with EPA and DHA contributes to the maintenance of normal visual and cognitive functions (EFSA Panel on Dietetic Products and Allergies 2010a,  2011). Here, although the molecular roles of EPA and DHA and clinical data agree in specific situations or conditions, such as premature babies, adolescents with ADHD and Alzheimer's patients, there is no clinical support for supplementation for the general population. For this reason, we believe that the health claims that EFSA allows in terms of vision and cognition, extended to the entire population, exceed the real need.

### Evidence suggest that DHA synthesis in healthy adults from ALA is sufficient to supply the brain

Scientific literature generally agrees that DHA synthesis in humans occurs with very low efficiency (Fig. 1). On average, the male appears to convert only 1% of ingested ALA to DHA in the liver (Goyens et al. 2006; Lin et al. 2010) and female 8% (Burdge and Wootton 2002). Companies that market EPA and DHA oils use this reported inefficiency in their marketing efforts, as it undoubtedly favours their business interests. Nevertheless, authors like Domenichiello and colleagues have elegantly argued that DHA synthesis in humans may be sufficient to maintain brain function (Domenichiello et al. 2015).

In this line, recent research suggests that prior evaluation of DHA synthesis in humans yielded equivocal results. Pignitter et al. have used circulating LDL (low-density lipoprotein) as a proxy to assess DHA synthesis in the liver, unlike previous studies, which looked for DHA in the fraction of circulating phospholipids, fatty acids or red blood cells. As a result, Pignitter et al. reported that in their experiment, 30% of ingested ALA was converted to DHA (Pignitter et al. 2018).

In addition to this, many studies have reported that the retina and brain bear cell types that synthesize DHA. Examples of this in the retina are the retinal pigment epithelium (Wang and Anderson 1993; Chen et al. 1999), microvascular endothelial cells (Delton-Vandenbroucke et al. 1997) and retinal neurons (Simón et al. 2016). In the brain, astrocytes (Moore et al. 1991; Williard et al. 2001), microvascular cells (Delton-Vandenbroucke et al. 1997) and some types of neurons (Kaduce et al. 2008) also produce DHA. A proper assessment of the magnitude of this DHA synthesized and used in the retina and brain could contribute to demystifying the low production capacity of DHA in humans.

Evolution also indicates that the synthesis of DHA in humans is a physiologically relevant process. The FADS1 and FADS2 genes are responsible for encoding the Δ-desaturases that constitute the bottlenecks that determine the synthesis rate of this fatty acid (Fig. 1). After 23 000 years of a diet of fish, seals and whales, the Inuit of Greenland have fixed less efficient alleles of FADS1 and FADS2 to produce less EPA and DHA and conserve ALA, scarce in their diet (Fumagalli et al. 2015). On the other hand, the predominantly vegetarian diet of Europeans during the Bronze Age caused them to fix more active alleles of FADS1 and FADS2 to produce more EPA and DHA (Buckley et al. 2017). The notion that the diet is capable of modulating the genetics of DHA synthesis throughout evolution suggests that this process is key in human physiology.

According to the above, despite the influence of diet on the levels of DHA in the blood [up to 40% less in vegans and vegetarians (Domenichiello et al. 2015)], there are factors such as gender, stage of life, genetic background and state of health that may have a determining role on internal levels of this fatty acid. For this reason, we believe that supplementation with EPA and DHA should be recommended only after a personalized analysis, and the EPA and DHA nutraceutical industry should not target healthy adults in their marketing efforts.

## Antarctic krill and *C. finmarchicus* fisheries. Management, threats to the ecosystem and climate change

Although supplementation with EPA and DHA seems unnecessary for healthy adults, we believe that this argument may be insufficient to stir consciences and reduce the exploitation of ecosystems to source these fatty acids. That is why we describe below how Antarctic krill and Calanus fisheries can add up to climate change and threaten unique ecosystems.

### *E. superba* and *C. finmarchicus*, ecological importance and commercial interest

**Fig. 3:**
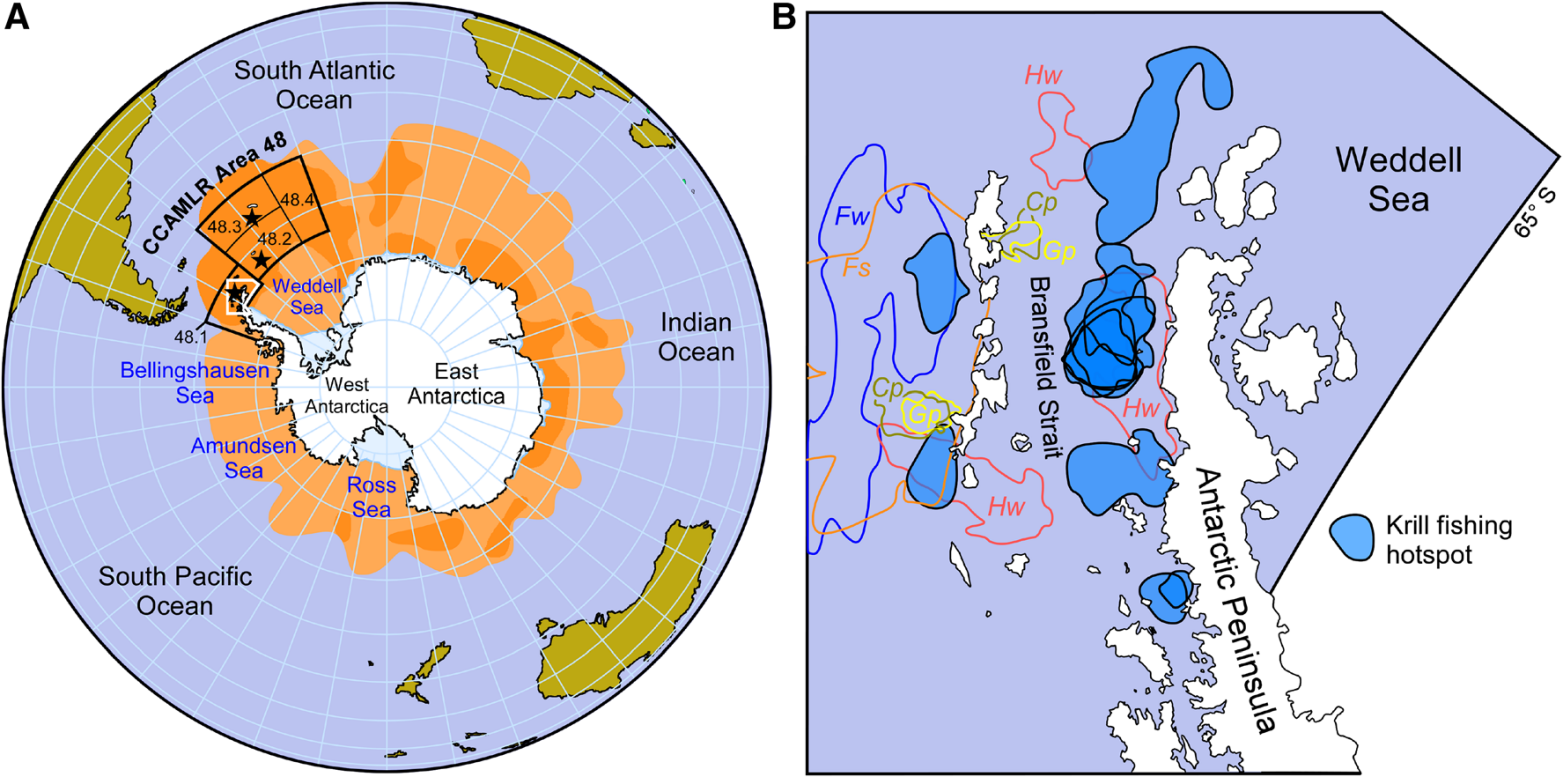
Density distribution of Antarctic Krill (*E. superba*) and current main fishing area of this crustacean (CCAMLR Statistical area 48), including fishing hotspots identified in Area 48.1. **a** Representation of the southern hemisphere, and distribution area of Antarctic krill, where the shaded areas of light or dark orange represent a low or high density of this crustacean, respectively (adapted from previously published data (Atkinson et al. 2004). The Antarctic krill fishing area currently mainly exploited (CCAMLR Area 48 and its subdivision in statistical subareas) is also shown. The black stars denote the fishing concentration zones, located in the South Shetland Islands in subarea 48.1, the South Orkney Islands in subarea 48.2 and South Georgia in subarea 48.3. The white bounded area represents the area displayed in (**b**). **b** Representation of the section of subarea 48.1 that contains the fishing hotspots identified by Santa Cruz et al. (2018). Each fishing hotspot is enclosed by a black border and is shaded in dark blue. Areas with borders in different colors represent the foraging areas of Antarctic krill predators such as humpback whales (*Hw*), Fin whales (*Fw*) (Herr et al. 2016), Chinstrap Penguins (*Cp*), Gentoo Penguins (*Gp*) (Miller et al. 2010) and Antarctic Fur Seals (*Fs*) (Hinke et al. 2017)

Antarctic krill, a crustacean often compared to shrimp and about 6 cm long, inhabits all waters surrounding Antarctica (Fig. 3), occupying the key intermediate trophic level of this ecosystem (Flores et al. 2012a). Here, this crustacean grazes phytoplankton and serves as feed for whales, penguins, seals, fish and birds (Murphy et al. 2007). Exploratory Antarctic fishing began in the early 1960s by the former Soviet Union, driven by declining high seas fish stocks and restricted access to the waters of coastal states (Hofman 2017). In 1977, R. M. Laws formulated what later became known as the 'krill surplus' hypothesis, according to which the observed increase in seal and penguin populations in those years was due to the previous near-extermination of the whales, which led to greater availability of krill for other species (Laws et al. 1977). At that time, this hypothesis was used by others to justify krill fisheries, since it was suggested that this ‘krill surplus’ could be destined for human use (Hofman 2017). Nevertheless, this hypothesis has been reviewed and considered incomplete (Surma et al. 2014). After capturing more than 400 000 tonnes of Antarctic krill per season in the 1980s and using it to feed pigs and chickens, to prepare food for humans or used as fertilizer (Nicol 2018), CCAMLR (Commission for the Conservation of Antarctic Marine Living Resources) entered into force to regulate this practice (CCAMLR 1980).

*C. finmarchicus* thrives in the subpolar waters of the North Atlantic Ocean (Scott et al. 2000), and is dominant in the Norwegian Sea (Choquet et al. 2017; Strand et al. 2020) (Fig. 5a). Likewise Antarctic krill, *C. finmarchicus* occupies the key intermediate level in its trophic web (Fauchald et al. 2011), grazing phytoplankton and serving as prey to the North Atlantic right whale (Cronin et al. 2017), and to fish species such as cod (in the larval stage) (Ottersen et al. 2014), herring (Prokopchuk 2009) and capelin (Buren et al. 2014). After small trials with this copepod for aquaculture, pet food and even as an ingredient for soups in the 1960s (Wiborg 1976), the company Calanus AS (Tromsø, Norway) started to fish this copepod experimentally in 2003 (Fiskeridirektoratet 2016). In 2016, the Norwegian Ministry of Climate and Environment (NMCE) regulated the commercial fishing of this copepod, and in 2019, this regulation entered into force (Nærings- og fiskeridepartementet 2019).

### Management rules of these fisheries

To protect Antarctic krill and its predators from the impact of the fishery, CCAMLR supervised an international effort to estimate krill biomass in the Atlantic sector of the Southern Ocean (Trathan et al. 1995), where 58–71% of total krill biomass exists (Atkinson et al. 2008), and the fishery was concentrating. CCALMR used this data together with the annual growth rate, mortality rate, and recruitment variability to calculate a precautionary fishing quota of 5.61 million tonnes per season, (9.23% of the estimated 60.3 million tonnes of krill biomass) (Butterworth et al. 1992; Constable et al. 2000; SC-CAMLR 2010). CCAMLR also established a lower quota known as trigger level to mark the use of more restrictive fishing rules under small-scale management units (SSMUs). The trigger level was established as the equivalent of the maximum annual catches in the 1980s, which were considered safe for the ecosystem at that time (SC-CAMLR 1991; Hill et al. 2016). This trigger level, set at 620 000 tonnes per season (roughly 1% of basal biomass), was divided among subareas 48.1–4 (Fig. 3) (CCAMLR 2016a). Once this volume of catches was reached in any of the subareas, the use of the SSMUs would begin. However, CCAMLR has not yet agreed on regulations for working with SSMUs (Nicol and Foster 2016), thus making the trigger level the interim catch limit.

**Fig. 4:**
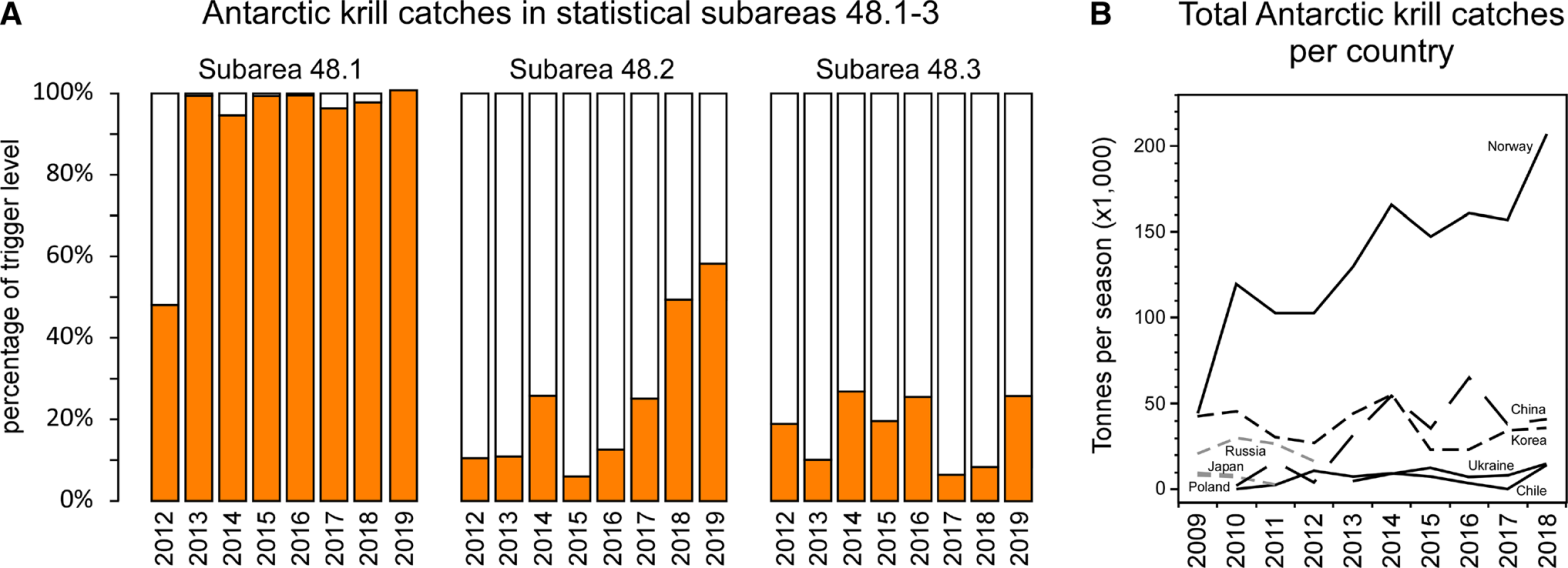
Catches of Antarctic krill. **a** Catches per season in statistical subareas 48.1, 48.2 and 48.3, represented as the percentage of the trigger level. Data obtained from the Fishery Report: *Euphasia superba* in Area 48 (CCAMLR Secretariat 2020). Each fishing season is represented by the year it ends.** b** Total seasonal catches per country. Data obtained from CCAMLR Statistical Bulletin (2019, Volume 31, Table 8.1—Catch Effort Data)

It has been suggested that the trigger level is not safe for the Antarctic ecosystem (Medley et al. 2009). This level has been reached in the last seven seasons in subarea 48.1, and captures are rapidly increasing in subarea 48.2 (Fig. 4a), driven by an increase in the activity of Norwegian companies (Fig. 4b) (CCAMLR 2019). Furthermore, fishing hotspots have been identified in subarea 48.1 (Fig. 3b) (Santa Cruz et al. 2018), which coincide with the feeding grounds of whales (Herr et al. 2016), penguins and Antarctic fur seals inhabiting this region (Miller et al. 2010; Hinke et al. 2017). A recent work by Watters and colleagues confirms the detrimental effect of these overlaps, finding a significant correlation of periods of penguin underperformance with years of high krill catches in their fishing grounds (Watters et al. 2020). Therefore, measures like the implementation of SSMU rules are urgent, but CCAMLR suffers a continuous internal struggle between the fishing and conservative interests of the countries that comprise this organisation (Jacquet et al. 2016; Hofman 2019). Other examples of this are the difficulties to establish marine protected areas (MPAs) (Brooks 2013; Brooks et al. 2020) or to address the impact of the fishery (Constable et al. 2000). Because of this, and because they are aware of the potential impact of their activities, fishing companies sometimes make unilateral decisions and, for example, temporarily suspend fishing near penguin colonies, even when CCAMLR has not reached an agreement to implement this measure (CCAMLR 2016b). Nevertheless, other movements of these companies are criticised, like the process of obtention of Certification as a Sustainable Fishery from the Marine Stewardship Council (MSC) by Aker BioMarine AS (Medley et al. 2009; Christian et al. 2013).

Despite the difficulties that CCAMLR faces in managing Antarctic krill fishery, the Norwegian Ministry of Climate and Environment (NMCE) has been inspired by the work of this organisation to establish a fishing quota for *C. finmarchicus*. Of note, although NMCE recognised the need for a research effort similar to that supervised by CCAMLR, this agency realised that obtaining this empirical data would require a long-term international initiative that was difficult to take on (Fiskeridirektoratet 2016). Of note, in 2012 the Norwegian Ministry of Fisheries and Coastal Affairs requested to the International Council for the Exploration of the Sea (ICES) an “exploratory assessment of *Calanus finmarchicus* in the Norwegian Sea”. However, this proposal was not supported financially, and ICES could only limit itself to commenting on the data generated by the Norwegian government. In its report, ICES noted that the estimated annual consumption of *C. finmarchicus* by pelagic and mesopelagic fish, as well as by invertebrates, left little (or no) biomass available for a fishery. In another comment, ICES focused on by-catch concerns, as this fishery uses 500-micron mesh nets that can collect fish eggs and larvae. Thus, in an experiment that caught 85 075 kg of *C. finmarchicus*, thousands of fish eggs and larvae were accidentally captured. In the worst-case scenario calculations, ICES estimated that this by-catch eliminated the possibility of recruitment of 41 724 cod, which is equivalent to 327 370 kg of fish. This is almost four times more biomass than that of captured *C. finmarchicus*. However, and as the authors ironically point out in their report, the value of Calanus oil is higher than that of cod (ICES 2017). The Norwegian government, for its part, considers this by-catch to be negligible, but encourages fishing to be limited to areas with low presence of fish eggs and larvae (Fiskeridirektoratet 2016).

NMCE estimated the basal biomass of *C. finmarchicus* in Norwegian waters at 33 million tonnes, and applied the precautionary fishing quota that CCAMLR calculated for Antarctic krill (9.23%, rounded to 10% of basal biomass) (Fiskeridirektoratet 2016). This yielded a precautionary catch limit of 3.3 million tonnes per season. As NMCE considered that there is uncertainty about the estimation of *C. finmarchicus* basal biomass, the resilience of this copepod and that of its predators against fishing, the effects of the accidental capture of fish eggs and the impact of climate change, this organism decided to adopt a more restrictive fishing quota. Again, NMCE, inspired by the work of CCAMLR, adopted their trigger level (1% of basal biomass) for *C. finmarchicus*. This yielded a catch limit of 330 000 tonnes per season (Fiskeridirektoratet 2016), which applied to 77% of the area surveyed, resulted in 254 000 tonnes of *C. finmarchicus* catch allowable per season (Nærings- og fiskeridepartementet 2019).

**Fig. 5:**
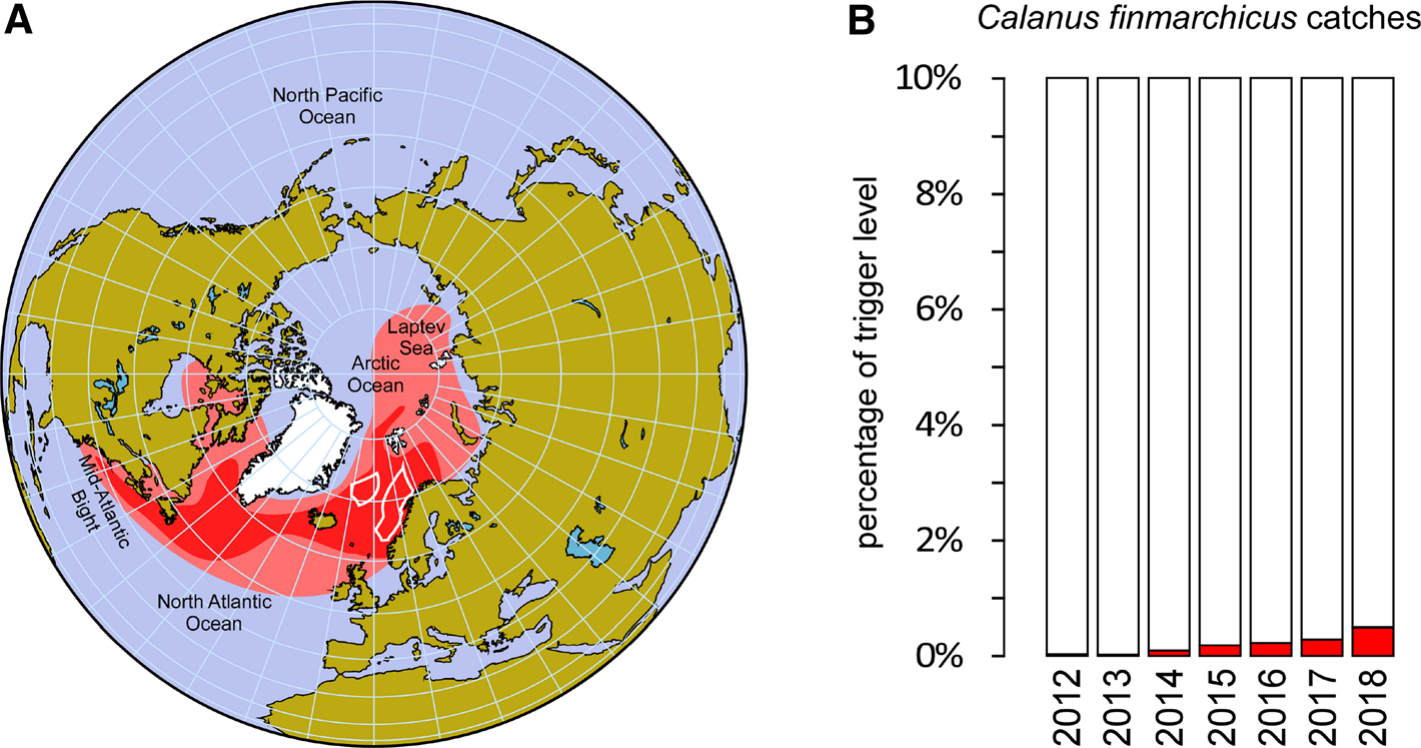
Density distribution of *C. finmarchicus*, fishing area and catches. **a** The light and dark red shaded areas represent the geographical distribution area of *C. finmarchicus*, adapted from (Choquet et al. 2017). The areas limited with white borders represent the commercial fishing areas of *C. finmarchicus* established by the Norwegian government until 2019, consisting of 50.6% of the survey area. **b** Total catch per season represented as the percentage of the trigger level (254 000 tonnes per season). Data provided by the Department for fisheries and aquaculture, Ministry of Trade, Industry and Fisheries, Norway

Until 2018, the catches of *C. finmarchicus* have been less than 1% of the trigger level (Fig. 5b). Therefore, at the current stage of development, this fishery is unlikely to have impacted the population of *C. finmarchicus* or its predators. However, the collateral capture of fish eggs and larvae in fishing nets is a cause of concern for the authorities and for the companies that fish for this crustacean. If this problem is not resolved, increased fishing efforts can affect the fish populations that inhabit the *C. finmarchicus* fishing area and, by extension, the species that feed on them (Eysteinsson et al. 2018). It is therefore surprising that NMCE uses CCAMLR rules for Calanus fishery when these rules are being questioned, and furthermore *C. finmarchicus* is a different species, lives in different waters and is part of a different trophic web.

### The impact of climate change and interactions with the fisheries

We have described above the weaknesses in the regulations that govern Antarctic krill and Calanus fisheries. Now we describe how climate change affects the exploited ecosystems and how the fisheries may aggravate this impact.

The area of Antarctic Peninsula is considered a climatological anomaly within Antarctica (Vaughan et al. 2003) since it is experiencing higher increases in surface air temperature than the rest of the continent (2–5 °C in the last 50 years) (Vaughan et al. 2001; Compo et al. 2011; Gonzàlez and Fortuny 2018). Water temperature increases in this area are also higher than average, due to a change in the wind regime that is causing a greater entry of deep circumpolar warm water in the Amundsen Sea (Cook et al. 2016; Holland et al. 2019). Thus, the increase in air and water temperatures are making the sea ice extent 7% lower (Comiso et al. 2011; Parkinson 2019) and last three days less each year (Stammerjohn et al. 2012). Glaciers are retreating (Cook et al. 2016), and ice shelves are melting (Etourneau et al. 2019; Rignot et al. 2019), which is promoting a decrease in the ice sheet of the Antarctic Peninsula (Shepherd et al. 2018; Rignot et al. 2019). Sea ice is a crucial habitat for the diatoms that constitute the main feed for the post-larval stage of krill (Montes-Hugo et al. 2009; Flores et al. 2012b). Although there is a debate about whether sea ice decline, water temperature increase and water acidification is affecting krill biomass (Atkinson et al. 2004; Melbourne-Thomas et al. 2016; Cox et al. 2018; Hill et al. 2019), a majority of researchers defend that climate change is threatening Antarctic krill (Saba et al. 2012; Kawaguchi et al. 2013; Piñones and Fedorov 2016), to a point that has forced a southward migration of this crustacean looking for colder waters (Atkinson et al. 2019).

Importantly, climate change and the fishery seem to be interacting to cause a decrease of penguins in the Antarctic Peninsula (Trivelpiece et al. 2011; Klein et al. 2018; Krüger et al. 2020) and produce stress in pack-ice seals (Forcada et al. 2012). Despite this, the moratorium on commercial whaling implemented in the mid-1980s is allowing the whales to recoup, with humpback whales (*Megaptera novaeanglae*) likely recovering their pre-exploitation levels by 2030 (Zerbini et al. 2019). Nevertheless, a rough estimate from these authors suggests that only humpback whales are currently removing 2.5–4.3% of Antarctic krill biomass per season in the South Atlantic. For its part, CCAMLR allows for captures up to 1% of Antarctic krill biomass in this sector of the Southern Ocean. If we also take into account the biomass of krill consumed by penguins, seals and birds, and the confluence of these species with the fishery in the same fishing grounds (Santa Cruz et al. 2018) there is a significant potential for a remodelling of the structure of this ecosystem (Zerbini et al. 2019). Furthermore, a more fierce fight for available krill, together with the expected decrease in biomass of this crustacean in the future due to climate change, has led different authors to predict that whale populations will decline again in the coming decades (Wiedenmann et al. 2011; Seyboth et al. 2016; Tulloch et al. 2019).

Sea surface temperature is also increasing in the North Atlantic Ocean (Abram et al. 2016), and *C. finmarchicus* does not seem able to adapt to this change (Hinder et al. 2014). As a consequence, the temperate species *C. helgolandicus* is replacing *C. finmarchicus* at the south of the Norwegian Sea (Montero et al. 2020), and this species is also decreasing in the south-western Norwegian Sea (Kristiansen et al. 2019). Ultimately, and similarly to Antarctic krill in the south, the distribution area of *C. finmarchicus* is shifting north (Beaugrand et al. 2002; Chust et al. 2013; Montero et al. 2020). The weakening of the Atlantic Southern Overturning Circulation (AMOC), also caused by global warming (Rahmstorf et al. 2015; Caesar et al. 2018; Thornalley et al. 2018) could result in less mobilisation of nutrients to the photic layer, which would negatively affect the microalgae blooms that *C. finmarchicus* feeds on (Osman et al. 2019). Decreases in the biomass of phytoplankton and changes in the timing of their blooms can, in turn, have a substantial impact on the blooms of *C. finmarchicus*. The specific lipid profile and lipid load of this species, as well as its size, are more advantageous for cod larvae than those of *C. helgolandicus*, the species that is replacing *C. finmarchicus* in these waters. These changes in the composition of the prey of cod larvae can result in a decrease in the survival and recruitment of this fish (Kattner and Hagen 2009; Kristiansen et al. 2011). Projections of a continuous scenario of high greenhouse gas emissions (temperature increase of about 4.3 °C by 2100, relative to pre-industrial temperatures) (IPCC 2013), predict that the biomass of *C. finmarchicus* may decrease up to 50% in the southern limits of its distribution at the end of this century (Grieve et al. 2017). These threats are expected to have cascade impacts on fish and the threatened North Atlantic right whale.

These whales, which mainly feed on this copepod (Baumgartner and Mate 2003; Pendleton et al. 2012), and of which only 409 individuals remain in the world (Pettis et al. 2019), may be irretrievably affected by these changes in prey availability (Meyer-Gutbrod and Greene 2018). Birds such as the common guillemot (*Uria aalge*) or the Atlantic puffin (*Fratercula arctica*), whose diet is based on fish that feed on *C. finmarchicus*, will entrust their survival to their adaptability to move northwards in search of prey (Frederiksen et al. 2013). Little auk (*Alle alle*), which feeds directly on *C. glacialis*, a more nutritious species than *C. finmarchicus* (Kidawa et al. 2014), could also be affected if this species is replaced by *C. finmarchicus* in northern waters (Karnovsky et al. 2010; Amélineau et al. 2019). These changes in the distribution and biomass of *C. finmarchicus* are also detrimental to its role in the sequestration of atmospheric carbon (Brun et al. 2019). Through its faecal pellets and discarded carapaces, as well as by the catabolism in the depths of the ocean of the fatty acids that they synthesize in the photic layer in spring, *C. finmarchicus* contributes to the role that the ocean plays in the control of atmospheric CO_2_ (Jónasdóttir et al. 2015).

## Concluding remarks

We tend to trust the publicity displayed by the companies commercialising nutritional supplements, either because we are not trained to challenge it, or we do not have time to evaluate all the points of view on their effectiveness. Besides, the possibility of consuming these products allows us a certain degree of self-indulgence with our lifestyle. Nevertheless, the best science available today suggests that supplements with EPA and DHA have no benefit in vascular or cognitive health for healthy adults. The best available science also conclude that climate change is affecting our planet, especially the poles and their ecosystems. Therefore, we encourage counteracting our comfortable position adopting a varied and healthy diet, practicing moderate exercise, and thinking that extracting EPA and DHA from our ecosystems threatens the equilibrium of the planet where we live.

For population groups that could benefit from supplementation, such as premature babies, children at risk for mental illness in adulthood due to genetically low levels of EPA and DHA, and for patients with Alzheimer's disease, we advocate for supplementation with EPA and DHA only by informed recommendation, and using non-extractive or recycled sources, such as the cultivation of heterotrophic microalgae or fish trimming.

We suggest EFSA and FDA re-evaluate the latest published meta-analyses to reconsider the health claims they allow. An eventual decrease in the importance of these health claims, together with truthful information about the threats that these fisheries and climate change pose, could push CCAMLR and the Norwegian government to apply the precautionary principle and work towards a gradual cessation of these fisheries. Importantly, we extend this request to forage fish fisheries, as these species, like Antarctic krill and *C. finmarchicus*, also occupy the key intermediate level in their trophic webs.

## References

[CR1] Abdelhamid AS, Brown TJ, Brainard JS, Biswas P, Thorpe GC, Moore HJ, Deane KHO, Summerbell CD (2020). Omega-3 fatty acids for the primary and secondary prevention of cardiovascular disease. Cochrane Database of Systematic Reviews.

[CR2] Abram, N.J., McGregor, H.V., Tierney, J.E., Evans, M.N., McKay, N.P., Kaufman, D.S., PAGES 2k Consortium, et al., 2016. Early onset of industrial-era warming across the oceans and continents. *Nature* 536, 411. 10.1038/nature19082 27558063

[CR3] Amélineau F, Grémillet D, Harding AMA, Walkusz W, Choquet R, Fort J (2019). Arctic climate change and pollution impact little auk foraging and fitness across a decade. Scientific Reports.

[CR4] Arca M, Borghi C, Pontremoli R, De Ferrari GM, Colivicchi F, Desideri G, Temporelli PL (2018). Hypertriglyceridemia and omega-3 fatty acids: Their often overlooked role in cardiovascular disease prevention. Nutrition, Metabolism and Cardiovascular Diseases.

[CR5] Astarita G, Jung K-M, Berchtold NC, Nguyen VQ, Gillen DL, Head E, Cotman CW, Piomelli D (2010). deficient liver biosynthesis of docosahexaenoic acid correlates with cognitive impairment in Alzheimer's disease. PLoS ONE.

[CR8] Atkinson A, Siegel V, Pakhomov E, Rothery P (2004). Long-term decline in krill stock and increase in salps within the Southern Ocean. Nature.

[CR9] Atkinson A, Siegel V, Pakhomov E, Rothery P, Loeb V, Ross R, Quetin LB, Schmidt K (2008). Oceanic circumpolar habitats of Antarctic krill. Marine Ecology Progress Series.

[CR6] Atkinson A, Hill SL, Barange M, Pakhomov EA, Raubenheimer D, Schmidt K, Simpson SJ, Reiss C (2014). Sardine cycles, krill declines, and locust plagues: Revisiting ‘wasp-waist’ food webs. Trends in Ecology & Evolution.

[CR7] Atkinson A, Hill SL, Pakhomov EA, Siegel V, Reiss CS, Loeb VJ, Steinberg DK, Schmidt K (2019). Krill (*Euphausia superba*) distribution contracts southward during rapid regional warming. Nature Climate Change.

[CR10] Aung T, Halsey J, Kromhout D, Gerstein HC, Marchioli R, Tavazzi L, Geleijnse JM, Rauch B (2018). Associations of omega-3 fatty acid supplement use with cardiovascular disease risks: Meta-analysis of 10 trials involving 77 917 individuals. JAMA Cardiology.

[CR11] Bang HO, Dyerberg J, Hjørne N (1976). The composition of food consumed by greenland Eskimos. Acta Medica Scandinavica.

[CR12] Barelli H, Antonny B (2016). Lipid unsaturation and organelle dynamics. Current Opinion in Cell Biology.

[CR13] Baumgartner M, Mate BR (2003). Summertime foraging ecology of North Atlantic right whales. Marine Ecology Progress Series.

[CR15] Beaugrand G, Reid PC, Ibañez F, Lindley JA, Edwards M (2002). Reorganization of North Atlantic marine copepod biodiversity and climate. Science.

[CR14] Beaugrand G, Brander KM, Alistair Lindley J, Souissi S, Reid PC (2003). Plankton effect on cod recruitment in the North Sea. Nature.

[CR16] Bhatt DL, Steg PG, Miller M, Brinton EA, Jacobson TA, Ketchum SB, Doyle RT, Juliano RA (2018). Cardiovascular Risk Reduction with Icosapent Ethyl for Hypertriglyceridemia..

[CR17] Bosch J, Gerstein HC, Dagenais GR, Díaz R, Dyal L, Jung H, Maggiono AP, Probstfield J (2012). n-3 fatty acids and cardiovascular outcomes in patients with dysglycemia. New England Journal of Medicine.

[CR18] Bowman L, Mafham M, Wallendszus K, Stevens W, Buck G, Barton J, Murphy K, Aung T (2018). Effects of n-3 fatty acid supplements in diabetes mellitus. New England Journal of Medicine.

[CR19] Bradbury J (2011). Docosahexaenoic acid (DHA): An ancient nutrient for the modern human brain. Nutrients.

[CR20] Brainard JS, Jimoh OF, Deane KHO, Biswas P, Donaldson D, Maas K, Abdelhamid AS, Hooper L (2020). Omega-3, Omega-6, and polyunsaturated fat for cognition: Systematic review and meta-analysis of randomized trials. J Am Med Dir Assoc.

[CR21] Brooks CM (2013). Competing values on the Antarctic high seas: CCAMLR and the challenge of marine-protected areas. The Polar Journal.

[CR22] Brooks CM, Chown SL, Douglass LL, Raymond BP, Shaw JD, Sylvester ZT, Torrens CL (2020). Progress towards a representative network of Southern Ocean protected areas. PLoS ONE.

[CR23] Brun P, Stamieszkin K, Visser AW, Licandro P, Payne MR, Kiørboe T (2019). Climate change has altered zooplankton-fuelled carbon export in the North Atlantic. Nature Ecology & Evolution.

[CR24] Buckley MT, Racimo F, Allentoft ME, Jensen MK, Jonsson A, Huang H, Hormozdiari F, Sikora M (2017). Selection in Europeans on fatty acid desaturases associated with dietary changes. Molecular Biology and Evolution.

[CR25] Burckhardt M, Herke M, Wustmann T, Watzke S, Langer G, Fink A (2016). Omega-3 fatty acids for the treatment of dementia. Cochrane Database of Systematic Reviews.

[CR26] Burdge GC, Wootton SA (2002). Conversion of alpha-linolenic acid to eicosapentaenoic, docosapentaenoic and docosahexaenoic acids in young women. British Journal of Nutrition.

[CR27] Buren AD, Koen-Alonso M, Pepin P, Mowbray F, Nakashima B, Stenson G, Ollerhead N, Montevecchi WA (2014). Bottom-up regulation of capelin, a keystone forage species. PLoS ONE.

[CR28] Burgoyne T, Meschede IP, Burden JJ, Bailly M, Seabra MC, Futter CE (2015). Rod disc renewal occurs by evagination of the ciliary plasma membrane that makes cadherin-based contacts with the inner segment. Proceedings of the National Academy of Sciences of the United States of America.

[CR29] Butterworth DS, Punt AE, Basson M (1992). A simple approach for calculating the potential yield of krill from biomass survey results. CCAMLR Science.

[CR30] Caesar L, Rahmstorf S, Robinson A, Feulner G, Saba V (2018). Observed fingerprint of a weakening Atlantic Ocean overturning circulation. Nature.

[CR31] Canhada S, Castro K, Perry IS, Luft VC (2018). Omega-3 fatty acids' supplementation in Alzheimer's disease: A systematic review. Nutritional Neuroscience.

[CR32] CCAMLR (1980). CCAMLR Convention Text.

[CR33] CCAMLR. 2016a. Conservation Measure 51-07. Interim distribution of the trigger level in the fishery for *Euphausia superba* in Statistical Subareas 48.1, 48.2, 48.3 and 48.4.

[CR34] CCAMLR. 2016b. Report of the Thirty-fifth Meeting of the Commission, Hobart, Australia.

[CR35] CCAMLR. 2019. CCAMLR Statistical Bulletin, Volume 31.

[CR200] CCAMLR Secretariat. 2020. Fishery Report: Euphausia superba in Area 48.

[CR36] Center for Food Safety and Applied Nutrition (CFSAN). 2014. RE: Petition for a Health Claim for Eicosapentaenoic Acid and Docosahexaenoic Acid and Reduction of Blood Pressure in the General Population (Docket No. FDA-2014-Q-1146) FDA.

[CR37] Chang JP-C, Su K-P, Mondelli V, Pariante CM (2018). Omega-3 polyunsaturated fatty acids in youths with attention deficit hyperactivity disorder: A systematic review and meta-analysis of clinical trials and biological studies. Neuropsychopharmacology.

[CR38] Chang JP-C, Su K-P, Mondelli V, Satyanarayanan SK, Yang H-T, Chiang Y-J, Chen H-T, Pariante CM (2019). High-dose eicosapentaenoic acid (EPA) improves attention and vigilance in children and adolescents with attention deficit hyperactivity disorder (ADHD) and low endogenous EPA levels. Translational Psychiatry.

[CR39] Chen H, Ray J, Scarpino V, Acland GM, Aguirre GD, Anderson RE (1999). Synthesis and release of docosahexaenoic acid by the RPE cells of PRCD-affected dogs. Investigative Ophthalmology & Visual Science.

[CR40] Choquet M, Hatlebakk M, Dhanasiri AKS, Kosobokova K, Smolina I, Søreide JE, Svensen C, Melle W (2017). Genetics redraws pelagic biogeography of Calanus. Biology Letters.

[CR41] Christian C, Ainley D, Bailey M, Dayton P, Hocevar J, LeVine M, Nikoloyuk J, Nouvian C (2013). A review of formal objections to marine stewardship council fisheries certifications. Biological Conservation.

[CR42] Chust G, Castellani C, Licandro P, Ibaibarriaga L, Sagarminaga Y, Irigoien X (2013). Are *Calanus* spp. shifting poleward in the North Atlantic? A habitat modelling approach. ICES Journal of Marine Science.

[CR43] Comiso JC, Kwok R, Martin S, Gordon AL (2011). Variability and trends in sea ice extent and ice production in the Ross Sea. Journal of Geophysical Research: Oceans.

[CR44] Compo GP, Whitaker JS, Sardeshmukh PD, Matsui N, Allan RJ, Yin X, Gleason BE, Vose RS (2011). The twentieth century reanalysis project. Quarterly Journal of the Royal Meteorological Society.

[CR45] Constable AJ, de la Mare WK, Agnew DJ, Everson I, Miller D (2000). Managing fisheries to conserve the Antarctic marine ecosystem: Practical implementation of the Convention on the Conservation of Antarctic Marine Living Resources (CCAMLR). ICES Journal of Marine Science.

[CR46] Cook AJ, Holland PR, Meredith MP, Murray T, Luckman A, Vaughan DG (2016). Ocean forcing of glacier retreat in the western Antarctic Peninsula. Science.

[CR47] Cox MJ, Candy S, de la Mare WK, Nicol S, Kawaguchi S, Gales N (2018). No evidence for a decline in the density of Antarctic krill *Euphausia superba* Dana, 1850, in the Southwest Atlantic sector between 1976 and 2016. Journal of Crustacean Biology.

[CR48] Cronin TW, Fasick JI, Schweikert LE, Johnsen S, Kezmoh LJ, Baumgartner MF (2017). Coping with copepods: Do right whales (*Eubalaena glacialis*) forage visually in dark waters?. Philosophical transactions of the Royal Society of London Series B, Biological Sciences.

[CR49] de Wilde MC, Vellas B, Girault E, Yavuz AC, Sijben JW (2017). Lower brain and blood nutrient status in Alzheimer's disease: Results from meta-analyses. Alzheimer's & Dementia (New York, N. Y.).

[CR50] Deane KHO, Jimoh OF, Biswas P, O'Brien A, Hanson S, Abdelhamid AS, Fox C, Hooper L (2019). Omega-3 and polyunsaturated fat for prevention of depression and anxiety symptoms: Systematic review and meta-analysis of randomised trials. The British Journal of Psychiatry.

[CR51] Delton-Vandenbroucke I, Grammas P, Anderson RE (1997). Polyunsaturated fatty acid metabolism in retinal and cerebral microvascular endothelial cells. Journal of Lipid Research.

[CR52] Do KV, Kautzmann M-AI, Jun B, Gordon WC, Nshimiyimana R, Yang R, Petasis NA, Bazan NG (2019). Elovanoids counteract oligomeric β-amyloid-induced gene expression and protect photoreceptors. Proceedings of the National Academy of Sciences of the United States of America.

[CR53] Domenichiello AF, Kitson AP, Bazinet RP (2015). Is docosahexaenoic acid synthesis from α-linolenic acid sufficient to supply the adult brain?. Progress in Lipid Research.

[CR54] Ducrocq F, Walle R, Contini A, Oummadi A, Caraballo B, van der Veldt S, Boyer ML, Aby F (2020). Causal link between n-3 polyunsaturated fatty acid deficiency and motivation deficits. Cell Metabolism.

[CR55] Dyerberg J, Bang HO, Hjørne N (1975). Fatty acid composition of the plasma lipids in Greenland Eskimos. The American Journal of Clinical Nutrition.

[CR56] EFSA Panel on Dietetic Products and Allergies. 2010a. Scientific Opinion on the substantiation of health claims related to docosahexaenoic acid (DHA) and maintenance of normal (fasting) blood concentrations of triglycerides (ID 533, 691, 3150), protection of blood lipids from oxidative damage (ID 630), contribution to the maintenance or achievement of a normal body weight (ID 629), brain, eye and nerve development (ID 627, 689, 704, 742, 3148, 3151), maintenance of normal brain function (ID 565, 626, 631, 689, 690, 704, 742, 3148, 3151), maintenance of normal vision (ID 627, 632, 743, 3149) and maintenance of normal spermatozoa motility (ID 628) pursuant to Article 13(1) of Regulation (EC) No 1924/2006. 8, 1734.

[CR57] EFSA Panel on Dietetic Products and Allergies. 2010b. Scientific Opinion on the substantiation of health claims related to eicosapentaenoic acid (EPA), docosahexaenoic acid (DHA), docosapentaenoic acid (DPA) and maintenance of normal cardiac function (ID 504, 506, 516, 527, 538, 703, 1128, 1317, 1324, 1325), maintenance of normal blood glucose concentrations (ID 566), maintenance of normal blood pressure (ID 506, 516, 703, 1317, 1324), maintenance of normal blood HDL-cholesterol concentrations (ID 506), maintenance of normal (fasting) blood concentrations of triglycerides (ID 506, 527, 538, 1317, 1324, 1325), maintenance of normal blood LDL-cholesterol concentrations (ID 527, 538, 1317, 1325, 4689), protection of the skin from photo-oxidative (UV-induced) damage (ID 530), improved absorption of EPA and DHA (ID 522, 523), contribution to the normal function of the immune system by decreasing the levels of eicosanoids, arachidonic acid-derived mediators and pro-inflammatory cytokines (ID 520, 2914), and “immunomodulating agent” (4690) pursuant to Article 13(1) of Regulation (EC) No 1924/2006. 8, 1796.

[CR59] EFSA Panel on Dietetic Products and Allergies (NDA). 2010. Scientific Opinion on Dietary Reference Values for fats, including saturated fatty acids, polyunsaturated fatty acids, monounsaturated fatty acids, trans fatty acids, and cholesterol. *EFSA Panel on Dietetic Products, Nutrition, and Allergies (NDA)* 8: 1461.

[CR58] EFSA Panel on Dietetic Products and Allergies. 2011. Scientific Opinion on the substantiation of health claims related to docosahexaenoic acid (DHA), eicosapentaenoic acid (EPA) and brain, eye and nerve development (ID 501, 513, 540), maintenance of normal brain function (ID 497, 501, 510, 513, 519, 521, 534, 540, 688, 1323, 1360, 4294), maintenance of normal vision (ID 508, 510, 513, 519, 529, 540, 688, 2905, 4294), maintenance of normal cardiac function (ID 510, 688, 1360), “maternal health; pregnancy and nursing” (ID 514), “to fulfil increased omega-3 fatty acids need during pregnancy” (ID 539), “skin and digestive tract epithelial cells maintenance” (ID 525), enhancement of mood (ID 536), “membranes cell structure” (ID 4295), “anti-inflammatory action” (ID 4688) and maintenance of normal blood LDL-cholesterol concentrations (ID 4719) pursuant to Article 13(1) of Regulation (EC) No 1924/2006. 9, 2078. 10.2903/j.efsa.2011.2078 PMC13129920 42078756

[CR60] Etourneau J, Sgubin G, Crosta X, Swingedouw D, Willmott V, Barbara L, Houssais M-N, Schouten S (2019). Ocean temperature impact on ice shelf extent in the eastern Antarctic Peninsula. Nature Communications.

[CR61] Eysteinsson ST, Gudjónsdóttir M, Jónasdóttir SH, Arason S (2018). Review of the composition and current utilization of *Calanus finmarchicus* – Possibilities for human consumption. Trends in Food Science & Technology.

[CR62] Fauchald P, Skov H, Skern-Mauritzen M, Johns D, Tveraa T (2011). Wasp–waist interactions in the north sea ecosystem. PLoS ONE.

[CR63] Fiskeridirektoratet H (2016). Forvaltningsplan for raudåte.

[CR64] Flores H, Atkinson A, Kawaguchi S, Krafft BA, Milinevsky G, Nicol S, Reiss C, Tarling GA (2012). Impact of climate change on Antarctic krill. Marine Ecology Progress Series.

[CR65] Flores H, van Franeker JA, Siegel V, Haraldsson M, Strass V, Meesters EH, Bathmann U, Wolff WJ (2012). The association of Antarctic Krill *Euphausia superba* with the under-ice habitat. PLoS ONE.

[CR66] Forcada J, Trathan PN, Boveng PL, Boyd IL, Burns JM, Costa DP, Fedak M, Rogers TL (2012). Responses of Antarctic pack-ice seals to environmental change and increasing krill fishing. Biological Conservation.

[CR67] Frederiksen M, Anker-Nilssen T, Beaugrand G, Wanless S (2013). Climate, copepods and seabirds in the boreal Northeast Atlantic—Current state and future outlook. Global Change Biology.

[CR68] Fumagalli M, Moltke I, Grarup N, Racimo F, Bjerregaard P, Jørgensen ME, Korneliussen TS, Gerbault P (2015). Greenlandic Inuit show genetic signatures of diet and climate adaptation. Science.

[CR69] Gasmi A, Mujawdiya PK, Shanaida M, Ongenae A, Lysiuk R, Doşa MD, Tsal O, Piscopo S (2020). *Calanus* oil in the treatment of obesity-related low-grade inflammation, insulin resistance, and atherosclerosis. Applied Microbiology and Biotechnology.

[CR70] Gawrisch K, Eldho NV, Holte LL (2003). The structure of DHA in phospholipid membranes. Lipids.

[CR71] GISSI-Prevenzione Investigators (1999). Dietary supplementation with n-3 polyunsaturated fatty acids and vitamin E after myocardial infarction: Results of the GISSI-Prevenzione trial. The Lancet.

[CR72] Gonzàlez S, Fortuny D (2018). How robust are the temperature trends on the Antarctic Peninsula?. Antarctic Science.

[CR73] Goyens PLL, Spilker ME, Zock PL, Katan MB, Mensink RP (2006). Conversion of α-linolenic acid in humans is influenced by the absolute amounts of α-linolenic acid and linoleic acid in the diet and not by their ratio. American Journal of Clinical Nutrition.

[CR74] Greenberg P (2018). The Omega principle: Seafood and the quest for a long life and a healthier planet.

[CR75] Grieve BD, Hare JA, Saba VS (2017). Projecting the effects of climate change on *Calanus finmarchicus* distribution within the U.S. Northeast Continental Shelf. Scientific Reports.

[CR76] Guo X-F, Li K-L, Li J-M, Li D (2019). Effects of EPA and DHA on blood pressure and inflammatory factors: A meta-analysis of randomized controlled trials. Critical Reviews in Food Science and Nutrition.

[CR77] Herr H, Viquerat S, Siegel V, Kock K-H, Dorschel B, Huneke WGC, Bracher A, Schröder M (2016). Horizontal niche partitioning of humpback and fin whales around the West Antarctic Peninsula: Evidence from a concurrent whale and krill survey. Polar Biology.

[CR78] Hilgendorf KI, Johnson CT, Mezger A, Rice SL, Norris AM, Demeter J, Greenleaf WJ, Reiter JF (2019). Omega-3 fatty acids activate ciliary FFAR4 to control adipogenesis. Cell.

[CR79] Hill S, Atkinson A, Darby C, Fielding S, Krafft B, Godø O, Skaret G, Trathan P (2016). Is current management of the Antarctic krill fishery in the Atlantic sector of the Southern Ocean precautionary?. CCAMLR Science.

[CR80] Hill SL, Atkinson A, Pakhomov EA, Siegel V (2019). Evidence for a decline in the population density of Antarctic krill *Euphausia superba* Dana, 1850 still stands. A comment on Cox et al. Journal of Crustacean Biology.

[CR81] Hinder SL, Gravenor MB, Edwards M, Ostle C, Bodger OG, Lee PLM, Walne AW, Hays GC (2014). Multi-decadal range changes vs. thermal adaptation for north east Atlantic oceanic copepods in the face of climate change. Global Change Biology.

[CR82] Hinke JT, Cossio AM, Goebel ME, Reiss CS, Trivelpiece WZ, Watters GM (2017). Identifying risk: Concurrent overlap of the Antarctic krill fishery with krill-dependent predators in the scotia sea. PLoS ONE.

[CR83] Hofman RJ (2017). Sealing, whaling and krill fishing in the Southern Ocean: Past and possible future effects on catch regulations. Polar Record.

[CR84] Hofman RJ (2019). Stopping overexploitation of living resources on the high seas. Marine Policy.

[CR85] Holland PR, Bracegirdle TJ, Dutrieux P, Jenkins A, Steig EJ (2019). West Antarctic ice loss influenced by internal climate variability and anthropogenic forcing. Nature Geoscience.

[CR86] ICES. 2017. *Report of the Working Group on Zooplankton Ecology (WGZE)*, 79. Copenhagen: International Council for the Exploration of the Sea.

[CR87] Imig J, Elmarakby A, Nithipatikom K, Wei S, Tuniki V (2010). Development of epoxyeicosatrienoic acid analogs with in vivo anti-hypertensive actions. Frontiers in Physiology.

[CR88] Stocker TF, Qin D, Plattner G-K, Tignor M, Allen SK, Boschung J, Nauels A, Xia Y, Bex V, Midgley PM, IPCC (2013). Summary for Policymakers. Climate change 2013: The physical science basis. Contribution of Working Group I to the Fifth Assessment Report of the Intergovernmental Panel on Climate Change.

[CR89] Jacquet J, Blood-Patterson E, Brooks C, Ainley D (2016). ‘Rational use’ in Antarctic waters. Marine Policy.

[CR90] Jenkins DJA, Sievenpiper JL, Pauly D, Sumaila UR, Kendall CWC, Mowat FM (2009). Are dietary recommendations for the use of fish oils sustainable?. CMAJ: Canadian Medical Association = Journal journal de l'Association medicale canadienne.

[CR91] Jónasdóttir SH, Visser AW, Richardson K, Heath MR (2015). Seasonal copepod lipid pump promotes carbon sequestration in the deep North Atlantic. Proceedings of the National Academy of Sciences of the United States of America.

[CR92] Kaduce TL, Chen Y, Hell JW, Spector AA (2008). Docosahexaenoic acid synthesis from n-3 fatty acid precursors in rat hippocampal neurons. Journal of Neurochemistry.

[CR93] Kantor ED, Rehm CD, Du M, White E, Giovannucci EL (2016). Trends in dietary supplement use among US adults from 1999–2012. JAMA.

[CR94] Karnovsky N, Harding MA, Walkusz W, Kwasniewski S, Goszczko I, Wiktor J, Rutti H, Bailey A (2010). Foraging distributions of little auks Alle alle across the Greenland Sea: Implications of present and future Arctic climate change. Marine Ecology Progress Series.

[CR95] Kattner G, Hagen W, Arts MT, Brett MT, Kainz M (2009). Lipids in marine copepods: Latitudinal characteristics and perspective to global warming. Lipids in aquatic ecosystem.

[CR96] Kawaguchi S, Ishida A, King R, Raymond B, Waller N, Constable A, Nicol S, Wakita M (2013). Risk maps for Antarctic krill under projected Southern Ocean acidification. Nature Climate Change.

[CR97] Kidawa D, Jakubas D, Wojczulanis-Jakubas K, Stempniewicz L, Trudnowska E, Boehnke R, Schönberger L, Blachowiak-Samolyk K (2014). Parental efforts of an Arctic seabird. Marine Biology Research.

[CR98] Klein ES, Hill SL, Hinke JT, Phillips T, Watters GM (2018). Impacts of rising sea temperature on krill increase risks for predators in the Scotia Sea. PLoS ONE.

[CR100] Kristiansen T, Drinkwater KF, Lough RG, Sundby S (2011). Recruitment variability in North Atlantic Cod and match-mismatch dynamics. PLoS ONE.

[CR99] Kristiansen I, Hátún H, Petursdottir H, Gislason A, Broms C, Melle W, Jacobsen JA, Eliasen SK (2019). Decreased influx of *Calanus* spp. into the south-western Norwegian Sea since 2003. Deep Sea Research Part I Oceanographic Research Papers.

[CR101] Krüger L, Huerta MF, Santa Cruz F, Cárdenas CA (2020). Antarctic krill fishery effects over penguin populations under adverse climate conditions: Implications for the management of fishing practices. Ambio.

[CR102] Lagarde M, Guichardant M, Bernoud-Hubac N, Calzada C, Véricel E (2018). Oxygenation of polyunsaturated fatty acids and oxidative stress within blood platelets. Biochimica et Biophysica Acta (BBA) - Molecular and Cell Biology of Lipids.

[CR103] Larqué E, Demmelmair H, Gil-Sánchez A, Prieto-Sánchez MT, Blanco JE, Pagán A, Faber FL, Zamora S (2011). Placental transfer of fatty acids and fetal implications. The American Journal of Clinical Nutrition.

[CR104] Laws RM, Fuchs VE, Maitland Laws R (1977). Seals and whales of the Southern Ocean..

[CR105] Liao Y, Xie B, Zhang H, He Q, Guo L, Subramaniapillai M, Fan B, Lu C (2019). Efficacy of omega-3 PUFAs in depression: A meta-analysis. Translational Psychiatry.

[CR106] Lin YH, Llanos A, Mena P, Uauy R, Salem N, Pawlosky RJ (2010). Compartmental analyses of (2)H(5)-α-linolenic acid and C-U-eicosapentaenoic acid toward synthesis of plasma labeled 22:6n–3 in newborn term infants. American Journal of Clinical Nutrition.

[CR107] Manni MM, Tiberti ML, Pagnotta S, Barelli H, Gautier R, Antonny B (2018). Acyl chain asymmetry and polyunsaturation of brain phospholipids facilitate membrane vesiculation without leakage. eLife.

[CR108] Manson JE, Cook NR, Lee IM, Christen W, Bassuk SS, Mora S, Gibson H, Albert CM (2019). Marine n-3 fatty acids and prevention of cardiovascular disease and cancer. New England Journal of Medicine.

[CR109] Martins BP, Bandarra NM, Figueiredo-Braga M (2020). The role of marine omega-3 in human neurodevelopment, including autism spectrum disorders and attention-deficit/hyperactivity disorder—A review. Critical Reviews in Food Science and Nutrition.

[CR110] Mason RP, Libby P, Bhatt DL (2020). Emerging Mechanisms of cardiovascular protection for the omega-3 fatty acid eicosapentaenoic Acid. Arteriosclerosis, Thrombosis, and Vascular Biology.

[CR111] McDougle DR, Watson JE, Abdeen AA, Adili R, Caputo MP, Krapf JE, Johnson RW, Kilian KA (2017). Anti-inflammatory ω-3 endocannabinoid epoxides. Proceedings of the National Academy of Sciences of the United States of America.

[CR112] Medley P, Pilling G, Payne A, Hough A, Davies S (2009). Aker Biomarine Antarctic krill pelagic trawl fishery certification.

[CR113] Melbourne-Thomas J, Corney SP, Trebilco R, Meiners KM, Stevens RP, Kawaguchi S, Sumner MD, Constable AJ (2016). Under ice habitats for Antarctic krill larvae: Could less mean more under climate warming?. Geophysical Research Letters.

[CR114] Messamore E, McNamara RK (2016). Detection and treatment of omega-3 fatty acid deficiency in psychiatric practice: Rationale and implementation. Lipids in Health and Disease.

[CR115] Meyer-Gutbrod EL, Greene CH (2018). Uncertain recovery of the North Atlantic right whale in a changing ocean. Global Change Biology.

[CR116] Miller AK, Kappes MA, Trivelpiece SG, Trivelpiece WZ (2010). Foraging-niche separation of breeding gentoo and chinstrap penguins, south shetland islands, Antarctica. The Condor.

[CR117] Montero JT, Lima M, Estay SA, Rezende EL (2020). Spatial and temporal shift in the factors affecting the population dynamics of Calanus copepods in the North Sea. Global Change Biology.

[CR118] Montes-Hugo M, Doney SC, Ducklow HW, Fraser W, Martinson D, Stammerjohn SE, Schofield O (2009). Recent changes in phytoplankton communities associated with rapid regional climate change along the Western Antarctic Peninsula. Science.

[CR119] Moore SA, Yoder E, Murphy S, Dutton GR, Spector AA (1991). Astrocytes, not neurons, produce docosahexaenoic acid (22:6ω-3) and arachidonic acid (20:4ω-6). Journal of Neurochemistry.

[CR120] Murphy EJ, Trathan PN, Watkins JL, Reid K, Meredith MP, Forcada J, Thorpe SE, Johnston NM (2007). Climatically driven fluctuations in Southern Ocean ecosystems. Proceedings: Biological Sciences.

[CR121] Nærings- og fiskeridepartementet. 2019. Forskrift om regulering av høsting av rødåte i 2019.

[CR122] Nicol S (2018). The curious Life of Krill. A conservation story from the bottom of the world.

[CR123] Nicol S, Foster J, Siegel V (2016). The fishery for Antarctic krill: Its current status and management regime. Biology and ecology of Antarctic krill.

[CR124] Noaghiul S, Hibbeln JR (2003). Cross-national comparisons of seafood consumption and rates of bipolar disorders. American Journal of Psychiatry.

[CR125] Norwegian Directorate of Fisheries. 2019. J-36-2019: Forskrift om regulering av høsting av rødåte i 2019 Norway.

[CR126] Ochiai Y, Uchida Y, Tachikawa M, Couraud P-O, Terasaki T (2019). Amyloid beta25-35 impairs docosahexaenoic acid efflux by down-regulating fatty acid transport protein 1 (FATP1/SLC27A1) protein expression in human brain capillary endothelial cells. Journal of Neurochemistry.

[CR127] Oh DY, Talukdar S, Bae EJ, Imamura T, Morinaga H, Fan W, Li P, Lu WJ (2010). GPR120 is an omega-3 fatty acid receptor mediating potent anti-inflammatory and insulin-sensitizing effects. Cell.

[CR128] Osman MB, Das SB, Trusel LD, Evans MJ, Fischer H, Grieman MM, Kipfstuhl S, McConnell JR (2019). Industrial-era decline in subarctic Atlantic productivity. Nature.

[CR129] Ottersen G, Bogstad B, Yaragina NA, Stige LC, Vikebø FB, Dalpadado P (2014). A review of early life history dynamics of Barents Sea cod (*Gadus morhua*). ICES Journal of Marine Science.

[CR130] Park HG, Park WJ, Kothapalli KSD, Brenna JT (2015). The fatty acid desaturase 2 (FADS2) gene product catalyzes Δ4 desaturation to yield n-3 docosahexaenoic acid and n-6 docosapentaenoic acid in human cells. The FASEB Journal.

[CR131] Parkinson CL (2019). A 40-y record reveals gradual Antarctic sea ice increases followed by decreases at rates far exceeding the rates seen in the Arctic. Proceedings of the National Academy of Sciences of the United States of America.

[CR132] Peet M (2004). International variations in the outcome of schizophrenia and the prevalence of depression in relation to national dietary practices: An ecological analysis. British Journal of Psychiatry.

[CR133] Pendleton DE, Sullivan PJ, Moira B, Cole TVN, Good CP, Mayo CA, Bruce M, Phillips S (2012). Weekly predictions of North Atlantic right whale *Eubalaena glacialis* habitat reveal influence of prey abundance and seasonality of habitat preferences. Endangered Species Research.

[CR134] Pettis, H.M., R.M. Pace, and P.K. Hamilton. 2019. North Atlantic Right Whale Consortium 2019 Annual Report Card. Report to the North Atlantic Right Whale Consortium.

[CR135] Pignitter M, Lindenmeier M, Andersen G, Herrfurth C, Beermann C, Schmitt JJ, Feussner I, Fulda M (2018). Effect of 1- and 2-month high-dose alpha-linolenic acid treatment on 13C-labeled alpha-linolenic acid incorporation and conversion in healthy subjects. Molecular Nutrition & Food Research.

[CR136] Piñones A, Fedorov AV (2016). Projected changes of Antarctic krill habitat by the end of the 21st century. Geophysical Research Letters.

[CR137] Prokopchuk I (2009). Feeding of the Norwegian spring spawning herring Clupea harengus (Linne) at the different stages of its life cycle. Deep Sea Research Part II: Topical Studies in Oceanography.

[CR138] Rahmstorf S, Box JE, Feulner G, Mann ME, Robinson A, Rutherford S, Schaffernicht EJ (2015). Exceptional twentieth-century slowdown in Atlantic Ocean overturning circulation. Nature Climate Change.

[CR139] Rice HB, Bernasconi A, Maki KC, Harris WS, von Schacky C, Calder PC (2016). Conducting omega-3 clinical trials with cardiovascular outcomes: Proceedings of a workshop held at ISSFAL 2014. Prostaglandins Leukotrienes and Essential Fatty Acids.

[CR140] Rignot E, Mouginot J, Scheuchl B, van den Broeke M, van Wessem MJ, Morlighem M (2019). Four decades of Antarctic Ice Sheet mass balance from 1979–2017. Proceedings of the National Academy of Sciences of the United States of America.

[CR141] Rizos EC, Ntzani EE, Bika E, Kostapanos MS, Elisaf MS (2012). Association between omega-3 fatty acid supplementation and risk of major cardiovascular disease events: A systematic review and meta-analysis. JAMA.

[CR142] Roncaglioni MC, Tombesi M, Avanzini F, Barlera S, Caimi V, Longoni P, Marzona I, Milani V (2013). n-3 fatty acids in patients with multiple cardiovascular risk factors. New England Journal of Medicine.

[CR143] Saba GK, Schofield O, Torres JJ, Ombres EH, Steinberg DK (2012). Increased feeding and nutrient excretion of adult Antarctic krill, *Euphausia superba*, exposed to enhanced carbon dioxide (CO_2_). PLoS ONE.

[CR144] Salas-Estrada LA, Leioatts N, Romo TD, Grossfield A (2018). Lipids alter rhodopsin function via ligand-like and solvent-like interactions. Biophysical Journal.

[CR145] Samadi M, Moradi S, Moradinazar M, Mostafai R, Pasdar Y (2019). Dietary pattern in relation to the risk of Alzheimer’s disease: A systematic review. Neurological Sciences.

[CR146] Santa Cruz F, Ernst B, Arata JA, Parada C (2018). Spatial and temporal dynamics of the Antarctic krill fishery in fishing hotspots in the Bransfield Strait and South Shetland Islands. Fisheries Research.

[CR147] SC-CAMLR. 1991. Report of the Tenth Meeting of the Scientific Committee (SC-CAMLR-X), p. 427, Hobart, Australia.

[CR148] SC-CAMLR. 2010. Report of the Fifth Meeting of the Subgroup on Acoustic Survey and Analysis Methods. In Report of the Twenty-ninth Meeting of the Scientific Committee (SC-CAMLRXXIX), Annex 5, pp. 147–171, Hobart, Australia.

[CR149] Scott CL, Kwasniewski S, Falk-Petersen S, Sargent JR (2000). Lipids and life strategies of *Calanus finmarchicus*, *Calanus glacialis* and *Calanus hyperboreus* in late autumn, Kongsfjorden, Svalbard. Polar Biology.

[CR150] Serhan CN, Levy BD (2018). Resolvins in inflammation: Emergence of the pro-resolving superfamily of mediators. The Journal of Clinical Investigation.

[CR151] Serini S, Calviello G (2020). Omega-3 PUFA responders and non-responders and the prevention of lipid dysmetabolism and related diseases. Nutrients.

[CR152] Seyboth E, Groch KR, Dalla Rosa L, Reid K, Flores PAC, Secchi ER (2016). Southern Right Whale (*Eubalaena australis*) reproductive success is influenced by krill (*Euphausia superba*) density and climate. Scientific Reports.

[CR153] Shahidi F, Ambigaipalan P (2018). Omega-3 polyunsaturated fatty acids and their health benefits. Annual Review of Food Science and Technology.

[CR154] Shepherd A, Ivins E, Rignot E, Smith B, van den Broeke M, Velicogna I, Whitehouse P, Briggs K (2018). Mass balance of the Antarctic ice sheet from 1992 to 2017. Nature.

[CR155] Shindou H, Koso H, Sasaki J, Nakanishi H, Sagara H, Nakagawa KM, Takahashi Y, Hishikawa D (2017). Docosahexaenoic acid preserves visual function by maintaining correct disc morphology in retinal photoreceptor cells. Journal of Biological Chemistry.

[CR156] Shulkin M, Pimpin L, Bellinger D, Kranz S, Fawzi W, Duggan C, Mozaffarian D (2018). n-3 Fatty acid supplementation in mothers, preterm infants, and term infants and childhood psychomotor and visual development: A systematic review and meta-analysis. The Journal of Nutrition.

[CR157] Simón MV, Agnolazza DL, German OL, Garelli A, Politi LE, Agbaga M-P, Anderson RE, Rotstein NP (2016). Synthesis of docosahexaenoic acid from eicosapentaenoic acid in retina neurons protects photoreceptors from oxidative stress. Journal of Neurochemistry.

[CR158] Simopoulos AP (2002). Omega-3 fatty acids and cardiovascular disease: The epidemiological evidence. Environmental Health and Preventive Medicine.

[CR159] Skinner ER, Watt C, Besson JA, Best PV (1993). Differences in the fatty acid composition of the grey and white matter of different regions of the brains of patients with Alzheimer's disease and control subjects. Brain.

[CR160] Spite M, Clària J, Serhan CN (2014). Resolvins, specialized proresolving lipid mediators, and their potential roles in metabolic diseases. Cell Metabolism.

[CR161] Stammerjohn S, Massom R, Rind D, Martinson D (2012). Regions of rapid sea ice change: An inter-hemispheric seasonal comparison. Geophysical Research Letters.

[CR162] Strand E, Bagøien E, Edwards M, Broms C, Klevjer T (2020). Spatial distributions and seasonality of four *Calanus* species in the Northeast Atlantic. Progress in Oceanography.

[CR163] Surma S, Pakhomov EA, Pitcher TJ (2014). Effects of Whaling on the structure of the Southern Ocean Food Web: Insights on the “Krill Surplus” from ecosystem modelling. PLoS ONE.

[CR164] Tavazzi L, Maggioni AP, Marchioli R, Barlera S, Franzosi MG, Latini R, Lucci D, Nicolosi GL (2008). Effect of n-3 polyunsaturated fatty acids in patients with chronic heart failure (the GISSI-HF trial): A randomised, double-blind, placebo-controlled trial. Lancet.

[CR165] Thornalley DJR, Oppo DW, Ortega P, Robson JI, Brierley CM, Davis R, Hall IR, Moffa-Sanchez P (2018). Anomalously weak Labrador Sea convection and Atlantic overturning during the past 150 years. Nature.

[CR166] Trathan PN, Everson I, Miller DGM, Watkins JL, Murphy EJ (1995). Krill biomass in the Atlantic. Nature.

[CR167] Trivelpiece WZ, Hinke JT, Miller AK, Reiss CS, Trivelpiece SG, Watters GM (2011). Variability in krill biomass links harvesting and climate warming to penguin population changes in Antarctica. Proceedings of the National Academy of Sciences of the United States of America.

[CR168] Tulloch VJD, Plagányi ÉE, Brown C, Richardson AJ, Matear R (2019). Future recovery of baleen whales is imperiled by climate change. Global Change Biology.

[CR169] Ursoniu S, Sahebkar A, Serban M-C, Antal D, Mikhailidis DP, Cicero A, Athyros V, Rizzo M (2017). Lipid-modifying effects of krill oil in humans: Systematic review and meta-analysis of randomized controlled trials. Nutrition Reviews.

[CR170] Vaughan DG, Marshall GJ, Connolley WM, King JC, Mulvaney R (2001). Devil in the detail. Science.

[CR171] Vaughan DG, Marshall GJ, Connolley WM, Parkinson C, Mulvaney R, Hodgson DA, King JC, Pudsey CJ (2003). Recent rapid regional climate warming on the Antarctic Peninsula. Climatic Change.

[CR172] Wang N, Anderson RE (1993). Synthesis of docosahexaenoic acid by retina and retinal pigment epithelium. Biochemistry.

[CR173] Wasserfurth P, Nebl J, Schuchardt JP, Müller M, Boßlau TK, Krüger K, Hahn A (2020). Effects of exercise combined with a healthy diet or *Calanus finmarchicus* oil supplementation on body composition and metabolic markers—A pilot study. Nutrients.

[CR174] Watson JE, Kim JS, Das A (2019). Emerging class of omega-3 fatty acid endocannabinoids & their derivatives. Prostaglandins & Other Lipid Mediators.

[CR175] Watters GM, Hinke JT, Reiss CS (2020). Long-term observations from Antarctica demonstrate that mismatched scales of fisheries management and predator–prey interaction lead to erroneous conclusions about precaution. Scientific Reports.

[CR176] Wiborg KF (1976). Fishery and commercial exploitation of *Calanus finmarchicus* in Norway. ICES Journal of Marine Science.

[CR177] Wiedenmann J, Cresswell KA, Goldbogen J, Potvin J, Mangel M (2011). Exploring the effects of reductions in krill biomass in the Southern Ocean on blue whales using a state-dependent foraging model. Ecological Modelling.

[CR178] Williard DE, Harmon SD, Kaduce TL, Preuss M, Moore SA, Robbins ME, Spector AA (2001). Docosahexaenoic acid synthesis from n-3 polyunsaturated fatty acids in differentiated rat brain astrocytes. Journal of Lipid Research.

[CR179] Yang H-J, Sugiura Y, Ikegami K, Konishi Y, Setou M (2012). Axonal gradient of arachidonic acid-containing phosphatidylcholine and its dependence on actin dynamics. Journal of Biological Chemistry.

[CR180] Yde Ohki CM, Grossmann L, Alber E, Dwivedi T, Berger G, Werling AM, Walitza S, Grünblatt E (2020). The stress–Wnt-signaling axis: A hypothesis for attention-deficit hyperactivity disorder and therapy approaches. Translational Psychiatry.

[CR181] Yokoyama M, Origasa H, Matsuzaki M, Matsuzawa Y, Saito Y, Ishikawa Y, Oikawa S, Sasaki J (2007). Effects of eicosapentaenoic acid on major coronary events in hypercholesterolaemic patients (JELIS): A randomised open-label, blinded endpoint analysis. The Lancet.

[CR182] Zehr KR, Walker MK (2018). Omega-3 polyunsaturated fatty acids improve endothelial function in humans at risk for atherosclerosis: A review. Prostaglandins & Other Lipid Mediators.

[CR183] Zerbini AN, Adams G, Best J, Clapham PJ, Jackson JA, Punt AE (2019). Assessing the recovery of an Antarctic predator from historical exploitation. Royal Society Open Science.

